# Role of exhaled nitric oxide as a predictor of atopy

**DOI:** 10.1186/1465-9921-14-48

**Published:** 2013-05-02

**Authors:** Karina M Romero, Colin L Robinson, Lauren M Baumann, Robert H Gilman, Robert G Hamilton, Nadia N Hansel, William Checkley

**Affiliations:** 1 Asociación Benéfica PRISMA, Lima, Peru; 2 CRONICAS Center of Excellence in Chronic Diseases, Universidad Peruana Cayetano Heredia, Lima, Peru; 3 Program in Global Disease Epidemiology and Control, Department of International Health, Bloomberg School of Public Health, Johns Hopkins University, Baltimore, USA; 4 Division of Allergy and Clinical Immunology, School of Medicine, Johns Hopkins University, Baltimore, USA; 5 Division of Pulmonary and Critical Care, School of Medicine, Johns Hopkins University, 1800 Orleans Street, Suite 9121, Baltimore, MD 21205, USA

**Keywords:** Allergic sensitization, Asthma, Exhaled nitric, Allergic rhinitis

## Abstract

**Background:**

The fractional exhaled nitric oxide (FeNO) is a quantitative, noninvasive and safe measure of airways inflammation that may complement the assessment of asthma. Elevations of FeNO have recently been found to correlate with allergic sensitization. Therefore, FeNO may be a useful predictor of atopy in the general population. We sought to determine the diagnostic accuracy of FeNO in predicting atopy in a population-based study.

**Methods:**

We conducted a cross-sectional study in an age- and sex- stratified random sample of 13 to 15 year-olds in two communities in Peru. We asked participants about asthma symptoms, environmental exposures and sociodemographics, and underwent spirometry, assessment of FeNO and an allergy skin test. We used multivariable logistic regression to model the odds of atopy as a function of FeNO, and calculated area-under-the-curves (AUC) to determine the diagnostic accuracy of FeNO as a predictor of atopy.

**Results:**

Of 1441 recruited participants, 1119 (83%) completed all evaluations. Mean FeNO was 17.6 ppb (SD=0.6) in atopics and 11.6 ppb (SD=0.8) in non-atopics (p<0.001). In multivariable analyses, a FeNO>20 ppb was associated with an increase in the odds of atopy in non-asthmatics (OR=5.3, 95% CI 3.3 to 8.5) and asthmatics (OR=16.2, 95% CI 3.4 to 77.5). A FeNO>20 ppb was the best predictor for atopy with an AUC of 68% (95% CI 64% to 69%). Stratified by asthma, the AUC was 65% (95% CI 61% to 69%) in non-asthmatics and 82% (95% CI 71% to 91%) in asthmatics.

**Conclusions:**

FeNO had limited accuracy to identify atopy among the general population; however, it may be a useful indicator of atopic phenotype among asthmatics.

## Background

The fractional exhaled nitric oxide (FeNO) is a non-invasive and sensitive biomarker of ongoing eosinophilic airway inflammation [1,2]. FeNO may be a useful marker in the assessment of asthma status and control [3,4]. It has shown potential promise as a non-invasive biomarker for asthma because it is a simple, well tolerated test with no risk to the participant [5] and it provides real-time, reproducible results in children aged ≥4 years [1-3,6-9]. For these reasons, FeNO has been recently recommended as a clinical endpoint for the characterization of study populations, in clinical trials and observational studies [10]. Recent studies, however, have reported high levels of FeNO even in well-controlled asthma [1,6,11], indicating that other factors may play a role in the determination of FeNO levels [12-15]. One potential factor that could explain variability in FeNO levels is atopic status.

Atopy is a clinical definition for an IgE-antibody responder, i.e., a personal tendency to become sensitized and produce IgE antibodies in response to allergens. Atopic individuals have an increased risk of developing asthma and other allergic diseases [16]. The definition of atopy, however, should only be considered when there is reported sensitization to allergen-specific IgE antibodies in serum or with a positive skin prick test to a specific allergen [16]. Recent studies have reported a strong association between FeNO and atopy [1,7,16,17]. Scott *et al.*[18] reported a positive correlation between FeNO and the number of positive skin prick tests in a cohort of asthmatics. Previous studies have also correlated aeroallergen sensitization with FeNO levels in atopic children [7,13,16,19,20]. These findings underscore the relevance of evaluating allergen sensitization status when FeNO is used as a biomarker in the diagnosis and monitoring of asthma [18]. More importantly, it also supports the hypothesis that FeNO may serve as a biomarker of atopy. The fact that atopy cannot always be identified using an allergy skin test [21], and the underlying risks involved in the determination of allergic skin sensitization increases the importance of studying the validity of FeNO as a simple, non-invasive biomarker for atopy [2,4,17].

The presence of a low to normal FeNO level in patients with chronic respiratory symptoms could also be helpful to rule out atopic status [20,22], however, there is a lack of strong evidence to support the role of FeNO in identifying atopy [2,5,11,23-25]. One recent study by Yao *et al.* reported that FeNO was a better marker of allergic sensitization than it was of asthma [17]. In this study, we seek to determine the clinical utility of FeNO as a non-invasive marker of atopy in a population-based study.

## Methods

### Study design

The study design is described in detail elsewhere [26]. We conducted a cross-sectional study of asthma prevalence in two regions in Peru. In December 2008, we selected a random sample of children aged 13 to 15 years from community censuses and visited them for enrollment into the study between April 2009 and December 2010. We asked participants about asthma and allergy symptoms, sociodemographics and environmental exposures, obtained anthropometry and a blood sample, and conducted an allergy skin test, a FeNO test and spirometry before and after bronchodilators. We used a previously validated Spanish version of the ISAAC questionnaire [13]. This study was approved by the Institutional Review Boards of the Johns Hopkins Bloomberg School of Public Health (Baltimore, USA) and A.B. PRISMA (Lima, Peru).

### FeNO assessment

We measured FeNO using a portable chemiluminescence analyzer (NIOXMINO, Aerocrine, Solna, Sweden) according to joint ERS/ATS recommendations [4,25]. No assessments were made if a participant reported a respiratory infection in the last 2 weeks or if the participant was on oral corticosteroids. We categorized FeNO levels using cut-off values of <20 ppb, 20-35 ppb and >35 ppb, respectively [25].

### Assessment of atopy

Allergy skin tests were performed using the Multi-Test II system (Lincoln Diagnostics, Decatur, IL) with allergen extracts made by ALK-Abello (Round Rock, TX). We used 10 allergens in the assessment: cockroach (*Blattella germanica*), dust mite mix (*Dermatophagoides farinae* and *D*. *pteronyssinus*), cat hair, dog epithelium, mouse epithelium, and mixed molds (*Alternaria, Cladosporium*, mixed *Aspergillus*, and mixed *Penicillium*). We also applied a histamine solution (10mg/ml) as a positive control and saline (0.9%) as a negative control. As per manufacturer’s instructions, we recorded vertical and horizontal measurements of induration and erythema, alongside 0–2 scales of itchiness and pseudopodia 20 minutes after application. Atopy was defined as a positive skin response to any of the allergen specificities as previously described [16,20,26].

### Definitions

We defined current asthma symptoms as wheeze or use of asthma medications in the past 12 months; allergic rhinitis as nasal symptoms (i.e., rhinorrhea, nasal discharge, nasal obstruction or nasal-ocular pruritus) without cold or flu symptoms in the past 12 months; and, smoking as self-reported tobacco use. We defined allergic symptoms if a child had either asthma symptoms or allergic rhinitis in the past 12 months. We calculated body mass index (BMI) percentile according to World Health Organization reference values [27]. We classified underweight as <5^th^ percentile; normal as 5^th^ to 84^th^ percentiles; overweight as 85^th^ to 94^th^ percentiles, and obese as ≥95^th^ percentile for their age and sex. We defined current inhaled corticosteroid use if the child used it in the last week.

### Biostatistical methods

We compared continuous variables between two subgroups with t-tests if normally distributed and with Wilcoxon rank-sum tests if not normally distributed, and compared dichotomous or categorical values between two subgroups with chi-square tests. We used multiple linear regression to identify risk factors associated with log-transformed FeNO in our study population. We used multiple logistic regression to estimate the odds of atopy for FeNO first as a continuous variable and then as a categorical variable using the above defined cut-offs, adjusted for sex, allergy symptoms, BMI, personal history of tobacco use, secondhand smoke, seasonality and site. We excluded current use of inhaled corticosteroids as a covariate because only 2 participants reported such intake. To assess the diagnostic accuracy of FeNO to predict atopy, we constructed receiver-operating-characteristic (ROC) curves and calculated the areas-under-the-curve (AUC) using five-fold cross validation [28]. We also conduced stratified analyses by asthmatic and rhinitis status. We conducted statistical analyses in STATA 11 (STATA Corp., College Station, USA).

## Results

### Characteristics of the study population

Of 1441 enrolled participants, 1199 (83%) completed both FeNO and allergy skin test assessments. We did not observe major differences between those with and without incomplete assessments (Table 1); however, we were more successful in obtaining both FeNO and an allergy skin tests among participants in Tumbes vs. Lima (p<0.001). Of the 1199 children with complete measurements, 52% were male, mean age was 14.9 years (SD=0.9), 21% were overweight, the prevalence of atopy was 46% based on skin test results and mean FeNO was 19.7 ppb (SD=22.6).

**Table 1:** Study Characteristics

**Variable**	**Children with FeNO and atopy data**	**Children with incomplete data**	**P**
Sample size	1199	242	
Male, % (n)	52% (622)	52% (125)	0.95
Age, mean (range)	14.9 (12.2-16.6)	14.8 (13.3-16.5)	0.43
Height in cm, mean (SD)	158.5 (8.2)	156.9 (7.4)	0.05
Current Asthma, % (n)	7% (89)	7.0% (17)	0.83
Allergic rhinitis, % (n)	17% (201)	21% (51)	0.11
Rural, % (n)	52% (625)	38% (91)	0.001
BMI, mean (range)	21.1 (13.9 – 39.2)	21.8 (15.1 – 36.6)	0.06
Secondhand smoke, % (n)	19% (232)	19% (46)	0.91
History of tobacco use, % (n)	5% (54)	4% (4)	0.59

### Factors associated with FeNO

Boys had higher FeNO levels than girls (15.8 ppb vs. 12.4 ppb; p<0.001). Children in Lima had higher FeNO levels than those in Tumbes (15.6 vs. 12.8 ppb; p<0.001). Mean FeNO was higher in atopics than in non-atopics (17.6 ppb vs. 11.6 ppb; p<0.001), higher in asthmatics than in non-asthmatics (27.9 ppb vs. 13.3 ppb; p<0.001) and higher in participants with allergic rhinitis compared to those without (21.8 ppb vs. 12.8; ppb p<0.001). FeNO also varied with season and personal history of tobacco use (all p<0.01). We did not find statistically significant differences in FeNO levels with tobacco smoke exposure, either personal or secondhand smoke or BMI categories (Table 2). In multiple linear regression with log FeNO as the outcome, important associations remained with atopy, current asthma symptoms, rhinitis and sex (p<0.001).

**Table 2:** Single variable and multivariable analyses of factors associated with Fractional exhaled nitric oxide (FeNO)

		**Fractional exhaled nitric oxide in ppb**			
	**n**	**Geometric mean (SD)**	**P**	**Coefficient in log ppb**	**P**
**Sex**					
Male	622	15.8 (0.7)	<0.001	Reference	
Female	577	12.4 (0.7)		−0.22	<0.001
**Atopy**					
Yes	556	17.6 (0.6)	<0.001	0.36	<0.001
No	643	11.6 (0.8)		Reference	
**Current asthma**					
Yes	89	27.9 (1.0)	<0.001	0.48	<0.001
No	1110	13.3 (0.7)		Reference	
**Allergic Rhinitis**					
Yes	201	21.8 (0.9)	<0.001	0.31	<0.001
No	998	12.8 (0.7)		Reference	
**Season of FeNO measurement**					
Fall	236	18.9 (21.6)	<0.01	Reference	
Winter	424	19.1 (22.9)		0.11	0.07
Spring	451	20.0 (23.3)		0.20	<0.001
Summer	88	23.1 (20.9)		0.23	0.01
**Site**					
Lima	574	15.6 (0.8)	<0.001	Reference	
Tumbes	625	12.4 (0.7)		−0.01	0.74
**Personal history of tobacco smoke**					
Yes	54	18.1 (0.8)	<0.01		
No	1086	14.1 (0.7)		0.12	0.20
**Body mass index (kg/m**^**2**^**)**					
Low weight	18	17.2 (15.9)	0.15	Reference	
Normal	936	18.9 (21.1)		0.01	0.93
Overweight	212	22.9 (23.2)		0.14	0.48
Obese	33	22.8 (28.7)		0.04	0.80
**Second hand smoke**					
Yes	232	14.3 (0.7)	0.11	−0.07	0.20
No	967	13.1 (0.8)		Reference	

### Predictors of atopy

In multivariable logistic regression, the odds of atopy increased with higher FeNO levels and with allergic symptoms, and was lower among children living in Tumbes vs. Lima (Table 3). We identified a dose–response relationship between FeNO and atopy (p<0.001). Having a FeNO>35 ppb was associated with atopy in children after adjusting for sex, height, BMI, site, allergy symptoms, personal history of tobacco, second hand smoke exposure and season of FeNO measurement (adjusted OR=5.6, 95% CI 3.6 to 8.8; p<0.001). FeNO levels 20–35 ppb were also associated with an increased odds of atopy (adjusted OR=2.2, 95% CI 1.5 to 3.1, p<0.001).

**Table 3:** Multivariable analyses of predictors of atopy in 1199 Peruvian children

**Variable**	**Crude Odds ratio**	**p-value**	**Adjusted Odds Ratio (95% CI)**	**p-value**
**Fractional exhaled nitric oxide**				
>35 ppb	6.7	<0.001	5.6 (3.7-8.8)	<0.001
20-35 ppb	2.5	<0.001	2.2 (1.5-3.2)	<0.001
<20 ppb	1.0		Reference	
**Site (rural)**	0.5	<0.001	0.4 (0.3-0.6)	<0.001
**Season of Fractional exhaled nitric oxide measurement**				
Fall	1.0		Reference	
Winter	0.5	<0.001	0.3 (0.2-0.5)	<0.001
Spring	0.4	<0.001	0.2 (0.2-0.4)	<0.001
Summer	1.1	0.861	0.4 (0.3-0.8)	<0.001
**Allergy Symptoms**	2.3	<0.001	1.5 (1.1-2.0)	0.03
**Body mass index**	1.1	0.01	1.0 (1.0-1.1)	0.27
**Personal smoke**	1.5	0.17	1.1 (0.6-2.0)	0.82
**Second hand smoke**	1.1	0.62	1.1 (0.8-1.6)	0.41

### Diagnostic accuracy of FeNO for atopy and asthma

**Figure 1:**
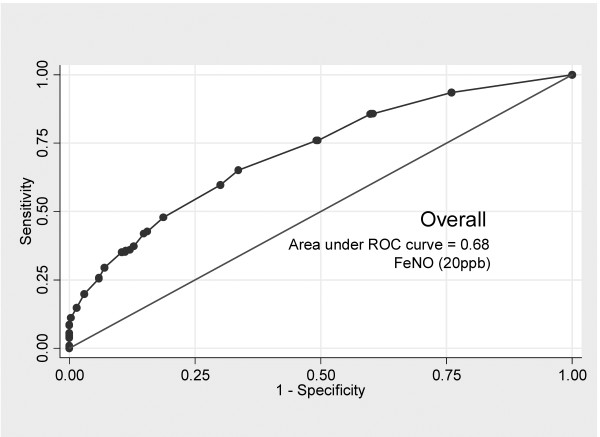
ROC Curve indicating the sensitivity and specificity of Fractional exhaled nitric oxide (FeNO) to predict atopy (FeNO > 20 ppb).

**Figure 2:**
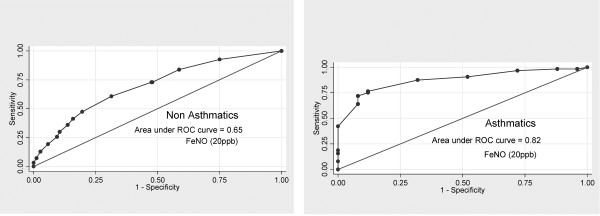
ROC Curve indicating the accuracy of Fractional exhaled nitric oxide (FeNO)measurements to predict atopy by asthma (FeNO > 20ppb).

We evaluated three different cut-off values of FeNO in relation to atopy, and found that FeNO>20 ppb was the best predictor for atopy (Table 4). Estimated AUC was 68% (95% CI 64% to 69%) after adjusting by sex, BMI, asthma, rhinitis and season of FeNO measurement (Figure 1). We observed better discrimination in specific subgroups. The AUC in non-asthmatics was only 65% (95% CI 61% to 69%), whereas it increased to 82% (95% CI 71% to 91%) in asthmatics (Figure 2). We also found that the AUC was 74% (95% CI 65% to 80%) and 66% (95% CI 62% to 70%) among those with and without allergic rhinitis, respectively.

**Table 4:** Diagnostic accuracy of Fractional exhaled nitric oxide (FeNO) for atopy

	**Sensitivity %**	**Specificity %**	**PPV %**	**NPV %**	**AUC% (95% CI)**
FeNO > 20 ppb	47.8	81.5	69.1	64.4	68 (64–69)
FeNO > 25 ppb	52.2	71.7	61.4	63.4	67 (64–70)
FeNO > 35 ppb	47.1	74.7	61.7	62.0	66 (63–69)
**By asthma status (FeNO > 20 ppb)**					
Non-asthmatics	37.7	84.8	66.1	62.9	65 (61–69)
Asthmatics	87.5	52.0	82.4	61.9	82 (71–91)

We found that there was gradient between the number of positive reactions and the prevalence of atopy. Specifically, mean FeNO for non-atopics was 14.2 ppb (SD=13.3); 17.0 ppb for atopics with 1 positive reaction (SD=15.4); 29.2 ppb (SD=33.0) for atopics with 2 positive reactions; and, 33.9 ppb (SD=33.6) for atopics with ≥3 positive reactions (p<0.001). The AUC increased from to 67% (95% CI 64% to 71%) to 73% (95% CI 70% to 76%) if we considered atopy in participants with ≥2 positive skin tests. Using similar analytical methods, we evaluated the same three FeNO cut-offs in relation to current asthma symptoms, and found that a FeNO>35 ppb had a AUC of 80% (95% CI 74% to 85%).

## Discussion

Our results suggest that FeNO had a modest ability to identify either atopy alone or asthma alone in our study population; however, our data suggest that it may be a useful aid in differentiating between atopic and non-atopic phenotypes among asthmatic children. We found that a FeNO>20 ppb may have sufficient discriminatory power to identify the asthmatic atopic phenotype.

Our results showed that FeNO had limited accuracy in identifying atopy in the general population. These findings contrast with those reported by Yao *et al.*[17], who reported a better discrimination of FeNO for allergic sensitization in the general population than that reported by our group (AUC of 80%, 95% CI 77% to 82%). Differences between the study conducted by Yao *et al.* and our current study could be attributed to the target population and assessment of atopy. Previous studies have described that age contributes to the variability of FeNO [4,5,8,18,20,25]. Our study had an older but narrower age range than the study by Yao *et al.* (5–18 years). Other studies suggest that FeNO may be more useful in young children, who often have no correlation with spirometric assessments or the manifestation of symptoms, but in whom a screening, early diagnosis, and preventive measures would be useful [19,20]. Other differences included the method of atopic assessment. Yao *et al.* conducted atopic assessment using the multi-allergen screen for serum specific IgE (e.g. Phadiatop), whereas we used allergy skin testing. Because allergy skin prick testing does not always identify atopy accurately, measurement of a panel of serum specific IgE is the best method to assess atopy. Some studies report a concordance between 85% and 95%, depending on the allergen being tested, between allergy skin testing and measurement of serum specific IgE [29]; however, it is still unclear if these two tests can be used interchangeably to determine atopic sensitization or if both should be used for the diagnosis of atopy [29-31]. Finally, another difference between both studies were that the study by Yao *et al.* and ours used different chemiluminescence analyzers for FeNO.

Variables such as sex, current asthma, allergic rhinitis, personal history of tobacco use, current use of inhaled corticosteroids, atopy and seasonality have all been previously identified as important explanatory factors that influence FeNO levels [17,20,24,32]. While our study corroborates the importance of these variables in our study setting, we also found that rural dwelling (i.e., living in Tumbes vs. Lima) was additional important explanatory factor associated with FeNO levels. Indeed, few studies have considered the rural versus urban setting in their analysis or study design, despite well-recognized differences in the prevalence of asthma and allergic disease between these two environments [26,33,34]. This is particularly relevant to investigations in low- and middle-income countries, as two recent studies conducted in South America, one in Ecuador [33] and another conducted by our team in Peru [26] have shown that urbanization increases the risk of both asthma and allergic diseases. The differences in FeNO levels are explained by the higher prevalence and increased severity of both asthma and atopy in Lima compared to Tumbes [26]. Use of tobacco could impact assessment of FeNO, and if under-reported, could have affected our results. We found an overall low prevalence of daily smokers in previous surveys of tobacco use in our study population. Using a previously-validated, Spanish questionnaires of tobacco smoke in the region, our group reported a low prevalence of daily smoking in adults [35].

Our findings may help to explain previous inconsistences that other studies have reported when using FeNO levels as a criterion in the diagnosis or management of asthma [6,8,12,15,22,23]. We found that FeNO>35 ppb predicted asthma with better accuracy than cutoffs of 20 ppb or 25 ppb. This points to the importance of proper characterization of the atopic phenotype when interpreting the relationship between FeNO and asthma, and also the proper consideration of particular cut-off FeNO values by atopic status, age and sex. Another explanation for previous inconsistences with other studies using FeNO levels could be related to methodology mostly related to flow dependence and the type of device used to measure FeNO. Recently, Malinovschi *et al.*[36] compared several methods of measuring FeNO and found a better association between asthma control using exhaled breath condensate nitrates rather than with chemiluminescence analyzers for FeNO, which is currently considered the gold standard [25,36,37].

Our study has some potential shortcomings. First, our findings are cross-sectional and we do not evaluate longitudinal changes in FeNO values within individuals. Studies have reported different coefficient of variations from 10% (about 4ppb) in healthy individuals to 40% in asthmatics [10-12]. Second, we assessed only at a narrow age range, and predictive cut-offs may change with age. Future investigations should include younger children or cover a broad age range, consider within-individual changes in FeNO levels and assessment of environmental allergenic exposures [10,21,25,34,37]. Third, we measured atopic sensitization to indoor aeroallergens only and did not include pollen or food allergens.

We chose not to measure pollens because Lima is located in a semi-arid, tropical region where there are few tree and grass allergens. While it is possible that food allergy may affect our overall prevalence of atopy and potentially the values of fractional exhaled nitric oxide [38]; however, the incidence of food allergies in our study population is unknown and understudied. Fourth, we did not conduct an evaluation of parasitic infections in our study children; however, previous population-based evaluations by our team on the burden of soil-based helminths in our study areas have been previously found to be low [39]. Finally, another aspect to consider is that potential genetic differences may exist between our study sites, which were settled by different ethnic groups; despite that phenotypically, these populations are similar (i.e., mestizo).

## Conclusion

In summary, our data suggest that FeNO had modest discriminatory power to identify atopy among the general population. It appeared to be a more useful tool to identify atopic phenotype among asthmatics. If this finding is further validated, FeNO may provide a simple, real-time non-invasive screen for atopy among asthmatics especially in resource-poor countries with limited access to medical specialists.

## Abbreviations

AUC: Area under the curve; PPV: Positive predictive value; NPV: Negative predictive value; CI: Confidence intervals; FeNO: Fractional exhaled nitric oxide; ppb: Parts per billion; ROC: Receiver operating characteristic.

## Competing interests

The authors have no conflicts of interest to disclose.

## Authors’ contributions

All authors participated in the study design and conduct, interpretation of findings and writing of manuscript. KR and WC conceived the study hypothesis, were primarily responsible for the analysis plan and writing of the manuscript. CR, LB, RG and NH were directly involved with study design and conduct. RH was involved in interpretation of results and provided critically important intellectual content. All authors read and approved the final manuscript.

## Authors’ information

**Other PURA study investigators include:** Juan Combe MD (A.B. PRISMA, Lima, Peru), Alfonso Gomez MD (A.B. PRISMA, Lima, Peru), Guillermo Gonzalvez MD (PAHO Lima, Peru), Lilia Cabrera RN (A.B. PRISMA, Lima, Peru), Robert Wise (Johns Hopkins University, Baltimore, USA), Kathleen Barnes PhD (Johns Hopkins University, Baltimore, USA), Patrick Breysse PhD (Johns Hopkins University, Baltimore, USA), D’Ann Williams PhD (Johns Hopkins University, Baltimore, USA).
